# Using Ancestry Informative Markers (AIMs) to Detect Fine Structures Within Gorilla Populations

**DOI:** 10.3389/fgene.2019.00043

**Published:** 2019-02-08

**Authors:** Ranajit Das, Ria Roy, Neha Venkatesh

**Affiliations:** ^1^Manipal Centre for Natural Sciences, Manipal Academy of Higher Education, Manipal, India; ^2^Department of Biotechnology Engineering, Sahrdaya College of Engineering and Technology, Kodakara, India; ^3^Department of Genetics, University of Mysore, Mysore, India

**Keywords:** ancestry informative marker (AIM), gorilla ancestry, conservation genetic management, admixture, informativeness of SNPs

## Abstract

The knowledge of ancestral origin is monumental in conservation of endangered animals since it can aid in preservation of population level genetic integrity and prevent inbreeding among related individuals. Despite maintenance of studbook, the biogeographical affiliation of most captive gorillas is largely unknown, which has constrained management of captive gorillas aiming at maximizing genetic diversity at the population level. In recent years, ancestry informative markers (AIMs) has been successfully employed for the inference of genomic ancestry in a wide range of studies in evolutionary genetics, biomedical research, genetic stock identification, and introgression analysis and forensic analyses. In this study, we sought to derive the AIMs yielding the most cohesive and faithful understanding of biogeographical affiliation of query gorillas. To this end, we compared three commonly used AIMs-determining methods namely, Infocalc, *F*_*ST*_, and Smart Principal Component Analysis (SmartPCA) with ADMIXTURE, using gorilla genome data available through Great Ape Genome Project database. Our findings suggest that the SNPs that were detected by at least three of the four AIMs-determining approaches (*N* = 1,531), is likely most suitable for delineation of gorilla AIMs. It recapitulated the finer structure within western lowland gorilla genomes with high degree of precision. We further have validated the robustness of our results using a randomized negative control containing the same number of SNPs. To the best of our knowledge, this is the first report of an AIMs panel for gorillas that may aid in developing cost-effective resources for large-scale demographic analyses, and greatly help in conservation of this charismatic mega-fauna.

## Background

Effective conservation of endangered animals with unknown ancestral origin entails delineation of the biogeographic affinities of their ancestors in order to facilitate preservation of the population level integrity of genomic signal. The knowledge of ancestral origin could be particularly relevant for planned re-introduction of animals to wild habitats and management of captive breeding programs in order to avoid inbreeding depression.

Gorillas, the largest living ape, were pronounced as critically endangered by IUCN Red List in 2007 (Walsh et al., 2008). Since the gorilla population is rapidly dwindling in the wild as a result of severe habitat encroachment and the illegal bushmeat trade, effective management of captive breeding programs has become monumental in order to both increase their numbers and to protect them from inbreeding. Overall 283 wild gorillas were imported to North America till 1970s, which subsequently stopped owing to the introduction of Convention on International Trade in Endangered Species of Wild Fauna and Flora (CITES) in 1975 (Nsubuga et al., 2010). It is noteworthy that despite maintenance of studbooks, insufficient information is available pertaining to the biogeographic origin of the majority of captive gorillas in the USA (Wharton, 2009) and that has likely constrained proper management of captive gorillas pertaining to maximizing genetic diversity at the population level. Proper knowledge of ancestry is of great importance in captive breeding programs of gorillas in order to avoid inbreeding depression and at the same time to conserve the genomic integrity of the native gorilla populations.

While whole genome approaches can efficiently resolve the biogeographical affiliation of gorillas by measuring genomic ancestry and level of admixture occurring among various gorilla populations, it is not cost-effective and dependent on the quality of DNA samples such that lower DNA quality (such as DNA extracted through non-invasive techniques) can hamper genome re-sequencing methods to a considerable extent. An alternative cost-effective strategy to whole genome approaches could be estimation of genomic ancestry using a handful of highly informative Single Nucleotide Polymorphisms (SNPs) which may range from a few hundreds to a few thousands. These highly informative SNPs that exhibit large differences in allele frequencies between ancestral populations are commonly referred to as Ancestry Informative Markers (AIMs) (Rosenberg et al., 2003; Shriver et al., 2003; Nassir et al., 2009).

Over the years AIMs panels have been successfully used for inferring biogeographical ancestry of humans (Rosenberg et al., 2003; Shriver et al., 2003; Kosoy et al., 2009; Nassir et al., 2009; Kidd et al., 2011; Tandon et al., 2011; Galanter et al., 2012; Huckins et al., 2014; Vongpaisarnsin et al., 2015), detection of illegal trade and translocation of wild animals (Frantz et al., 2006), food forensics (Wilkinson et al., 2012), genetic stock identification and introgression analysis (Munoz et al., 2015), forensic analysis (Phillips et al., 2016) to name a few. Recently, 9,000 genetic markers have been identified which are unique to a specific subspecies of chimpanzee and gorilla, and around 40,000 markers have been detected that are specific to each hominoid species or lineage (Hormozdiari et al., 2013).

In this study, we have compared three strategies previously used for AIMs determination, namely Infocalc algorithm (Paschou et al., 2007; Kosoy et al., 2009), Wright's *F*_ST_ (Tian et al., 2007; Kidd et al., 2011; Nievergelt et al., 2013), Smart Principal Component Analysis (SmartPCA) (Patterson et al., 2006) with a novel ADMIXTURE based approach (Alexander et al., 2009) to interrogate previously published whole genome data of 31 gorillas available in Great Ape Genome Project (GAGP) (Prado-Martinez et al., 2013) corresponding to two subspecies of western gorillas (*Gorilla gorilla*), namely western lowland gorilla (*Gorilla gorilla gorilla*) and Cross River gorilla (*Gorilla gorilla dielhi*), as well as the eastern lowland gorilla (*Gorilla beringei graueri*), to delineate an AIMs panel that can reproducibly capture the genomic ancestry of gorillas at the population level and aid in identification of gorillas at the individual level.

We performed our analysis in three steps. In the first step we evaluated the performance of the four AIMs determining approaches (Wright's *F*_*ST*_, Infocalc, SmartPCA and ADMIXTURE) by comparing them with complete SNP sets (CSS). Subsequently, we developed a consensus dataset, incorporating the SNPs that are common to at least three of the four AIMs-determining strategies. Finally, we developed a negative control dataset (randomly chosen SNPs from CSS) containing the same number of SNPs as the consensus dataset and re-evaluated the performance of the consensus dataset and four AIMs determining approaches. The consideration of the consensus SNPs as the AIMs panel for gorilla was robust since it balanced out the limitations of each individual AIMs determining method and at the same time recapitulated the ancestry information of query gorillas with high precision.

## Methods

### Dataset

The dataset employed in this study comprised of 31 gorilla genomes available in GAGP, which overall sequenced 79 great ape individuals to a mean coverage of 25X in an Illumina HiSeq 2000 platform (Prado-Martinez et al., 2013; Das and Upadhayai, 2018): western lowland gorilla (*Gorilla gorilla gorilla, N* = 27), eastern lowland gorilla (*Gorilla beringei graueri, N* = 3), and Cross River gorilla (*Gorilla gorilla dielhi, N* = 1). As indicated previously (Prado-Martinez et al., 2013; Das and Upadhayai, 2018) the western lowland gorilla genomes employed in this study belong to three distinct wild populations: Cameroonian, Congolese, and Equatorial Guinean. The biogeographical origin of the gorilla genomes as mentioned in the Studbook and that predicted through Geographical Population Structure (GPS) algorithm is mentioned in Supplemental Table 1. The same dataset comprised of 354,080 markers that has been used recently for tracing ancestry of gorillas (Das and Upadhayai, 2018) was used in this study.

### Population Clustering and Admixture Analysis Employing the CSS

Principal component analysis (PCA) was performed in PLINK v1.9 using - -pca command. The ancestry of the gorilla genomes was estimated using unsupervised clustering as implemented in ADMIXTURE v1.3 (Alexander et al., 2009). Similar to our recent study (Das and Upadhayai, 2018), we chose *K* = *3* for all downstream analysis to differentiate the western gorilla genomes into the Congolese and Cameroonian clusters and detection of AIMs for identification of genomic ancestry of gorillas at the population level. PCA and Admixture plots were generated in R v3.2.3.

### Determination of AIMs

In order to deduce the SNP markers that are able to infer the genomic ancestry of gorilla samples with accuracy comparable to that of the CSS of 354,080 SNPs, we evaluated four AIMs determining approaches enumerated below.

#### 1. Infocalc

The first method employed was the Infocalc algorithm (Rosenberg et al., 2003), implemented in Infocalc v1.1, which determines the amount of information multiallelic markers provide regarding an individual's ancestry by calculating the informativeness (*I*) of each marker individually. Infocalc determines *I* based on the mathematical expression described previously (Rosenberg et al., 2003):

$$\begin{array}{l} I=\overset{N}{\underset{j=1}{\sum }}\left(-p_{j}\log p_{j}+\overset{K}{\underset{i=1}{\sum }}\frac{p_{ij}}{K}\log p_{ij}\right) \end{array}$$

Where, *p*_*j*_ is the mean frequency of allele *j* over all populations, *p*_*ij*_ is the relative frequency of allele *j* in population *i* and *K* is the total number of populations.

We selected the top 10,000 most informative markers from the Infocalc v1.1 output file. Infocalc v1.1 compatible files were generated by using - -structure modifier to the PLINK v1.9 command line. The top 10,000 most informative markers were selected based on the informativeness defining column (*I_n*) of the output file (Supplemental Figure S1).

#### 2. Wright's *F_*ST*_*

*F*_*ST*_ (Sewall Wright, 2006) measures the degree of differentiation among populations likely arising due to genetic structure within them. Given a set of populations, PLINK estimated the fixation indices (*F*_*ST*_) separately for all 354,080 markers under evaluation in this study using - -*Fst* command. The Family ID (FID) was used as the indicator of the geographical affinity of the gorilla genomes to different wild populations as mentioned previously (Prado-Martinez et al., 2013) and/or estimated through our recent biogeographical analysis (Das and Upadhayai, 2018).

The 10,000 SNPs with highest *F*_*ST*_ values were selected for subsequent analyses (Supplemental Figure S2).

#### 3. ADMIXTURE

Analyzing the ADMIXTURE output file with SNP information (P file) for *K* of 3, we identified 10,662 SNPs with high *K* (column to column) variance (≥ 0.15).

#### 4. SmartPCA

In order to determine the most informative markers, SNP weightings for each principal component (PC) were calculated using the “SmartPCA” algorithm implemented in EIG v7.2.1 (Patterson et al., 2006; Price et al., 2006). SmartPCA, which is especially designed for analysis of genomic data, employs PCA to determine whether the test samples come from one homogenous population or there is any signature of population structure and outputs principal components (eigenvectors) and eigenvalues. In addition to these two files SmartPCA generates a “snpwt” file, depicting the weight of all 354,080 markers for each principal component.

The 10,000 SNPs with the highest “weights” for the first principal component (PC1) was selected for subsequent analyses (Supplemental Figure S3).

### Estimation of Candidate AIMs Panels

To determine the optimal AIMs-determining strategy for gorilla genomes, we first compared the datasets comprising of the top 10,000 SNPs generated through *F*_ST_, Infocalc, and SmartPCA with 10,662 SNPs detected through ADMIXTURE both qualitatively (via Admixture analysis and PCA) and quantitatively (by computing the Euclidean distances between the admixture components of the query datasets and the CSS).

Further we developed a consensus dataset, containing SNPs that are common to the four AIMs determining strategies (*F*_ST_, Infocalc, Admixture, and SmartPCA-based). Here, we note that only 37 SNPs were found to be common to all four approaches evaluated in this study, which was insufficient to recapitulate intraspecific ancestry information of the query gorillas (data not shown). So, in order to generate a consensus SNP panel that is likely to be sufficient to detect the fine structure within western gorilla populations, we developed a dataset comprising of 1,531 SNPs that were common to at least three of the four AIMs-determining methods (Supplemental Figure S4). Finally, to adjudge the predictive accuracy of the candidate AIMs datasets, we developed a negative control dataset by randomly sampling 1,531 SNPs from CSS and compared this with those comprising of the top 1,531 SNPs extracted through *F*_ST_, Infocalc, Admixture, SmartPCA-based methods and the consensus.

## Results

### ADMIXTURE Analyses

#### Qualitative Analysis

**Figure 1:**
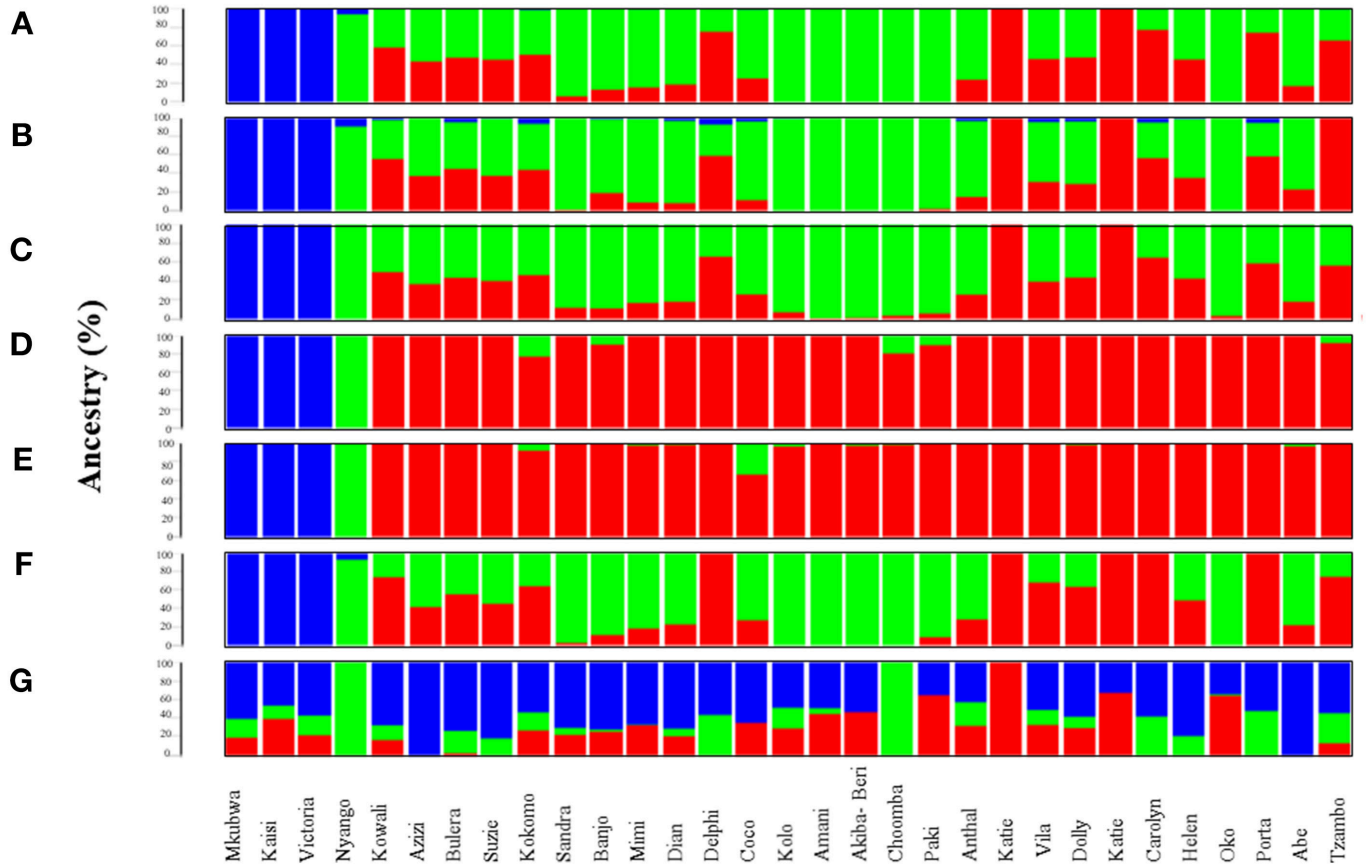
Admixture analysis of data subsets generated through top 1,531 most informative SNPs detected by various AIMs-determining strategies. Admixture plots showing the ancestry components of gorilla genomes. **(A)** Admixture analysis of the CSS (354,080 SNPs); **(B)** Admixture analysis of Infocalc-1,531 dataset; **(C)** Admixture analysis of Admixture-1,531 dataset; **(D)** Admixture analysis of SmartPCA-1,531 dataset; **(E)** Admixture analysis of *F*_*ST*_-1,531 dataset; **(F)** Admixture analysis of Consensus-1,531 dataset; and **(G)** Admixture analysis of Random-1,531 dataset. Admixture proportions were generated through an unsupervised admixture analysis at *K* = 3 using ADMIXTURE v1.3 and plotted in R v3.2.3. Each individual is represented by a vertical line partitioned into colored segments whose lengths are proportional to the contributions of the ancestral components to the genome of the individual. Blue represents eastern lowland ancestry component while green and red represent Cameroonian and Congolese ancestral components, respectively.

The ancestry of 31 gorilla genomes was estimated using unsupervised clustering as implemented in ADMIXTURE v1.3 (Alexander et al., 2009). For CSS, at *K* = 3 the eastern lowland gorillas were homogeneously assigned to a unique cluster (blue) while most western gorillas appeared to be a genomic admixture of Cameroonian (green) and Congolese (red) components in varying proportions (Figure 1A, Supplemental Figure S5A). While the entire genome of Akiba-Beri, Choomba, Paki, Oko, Kolo and Amani is consisted of the Cameroonian admixture component, Katie (B650) and Katie (KB4986) also appeared to be pure-bred and their genome is entirely composed of the Congolese admixture component.

At *K* = 3, the dataset comprising of the top 10,000 Inforcalc SNPs (Infocalc-10,000) performed the best by successfully and precisely capturing the population structure of gorilla genomes as depicted by the CSS. It homogenously assigned Akiba-Beri, Choomba, Paki, Oko, Kolo and Amani to Cameroon and the Katies (B650 and KB4986) to Congo. Further, similar to the CSS, this dataset revealed fractions of eastern lowland ancestry (blue) in Kokomo, Mimi, Delphi, Coco, Carolyn, and Porta. However, unlike the CSS, Infocalc-10,000 revealed minor fractions of (<1%) eastern lowland ancestry in Kowali and Azizi (Figure 1B, Supplemental Figure S5B).

The dataset comprising of the top 10,662 Admixture SNPs (Admixture-10,000) appeared to be the second best. In concordance with CSS, Admixture-10,000 homogenously assigned Akiba-Beri, Choomba, Oko and Amani to Cameroon and the Katies (B650 and KB4986) to Congo. However, unlike the CSS, this dataset depicted ~2, 3, and 4% Congolese ancestral component in the cross river gorilla Nyango, Kolo and Paki, respectively, and eastern lowland ancestral component in Helen and Anthal, which can be attributed to the likely loss of resolution (Supplemental Figure S5C).

The remaining two datasets, comprising of 10,000 SNPs generated using SmartPCA and *F*_*ST*_-based approaches (SmartPCA-10,000 and *F*_*ST*_-10,000, respectively), performed moderately. While SmartPCA-10,000 successfully homogenously assigned Akiba-Beri, Choomba, Paki, Oko, Kolo and Amani to Cameroon and the Katies (B650 and KB4986) to Congo, it additionally assigned Delphi, Carolyn and Porta homogenously to Congo and thus failed to capture their discernible proportions of Cameroonian ancestry (Supplemental Figure S5D). Among the four approaches, *F*_*ST*_-10,000 performed the worst. In addition to incorrectly assigning Delphi, Carolyn and Porta homogenously to Congo, *F*_*ST*_-10,000 revealed Congolese ancestry in Kolo, Akiba-Beri and Paki, which were otherwise homogenously assigned to Cameroon by all AIMs-determining approaches (Supplemental Figure S5E).

Among datasets comprising of top 1,531 SNPs deduced via *F*_ST_, Infocalc, Admixture, and SmartPCA, the 1,531 SNPs derived using Infocalc (Infocalc-1,531) was superior to the rest and most comparable to the CSS in recapitulating the population structure for query gorillas (Figure 1B). This was closely followed by a panel of 1,531 SNPs generated as a consensus of at least three of the four AIMs-determining strategies (Consensus-1,531) (Figure 1F), and that were detected using Admixture (Admixture-1,531) (Figure 1C). Here we note that among all 1,531 datasets, only Consensus-1,531 and Infocalc-1,531 were the only two who could capture the eastern lowland ancestry in the cross river gorilla, Nyango, as revealed by the CSS. In contrast, the SNP panel inferred using SmartPCA (SmartPCA-1,531) and *F*_*ST*_ (*F*_*ST*_-1,531) completely failed to capture the population structure revealed by the CSS (Figures 1D,E). Finally, the negative control dataset comprising of 1,531 random SNPs (Random-1,531) was expectedly unsuccessful in capturing the ancestry information of the query gorillas, underscoring the superiority of the AIMs over randomly selected markers in delineating ancestry information (Figure 1G).

#### Quantitative Analysis

**Figure 2:**
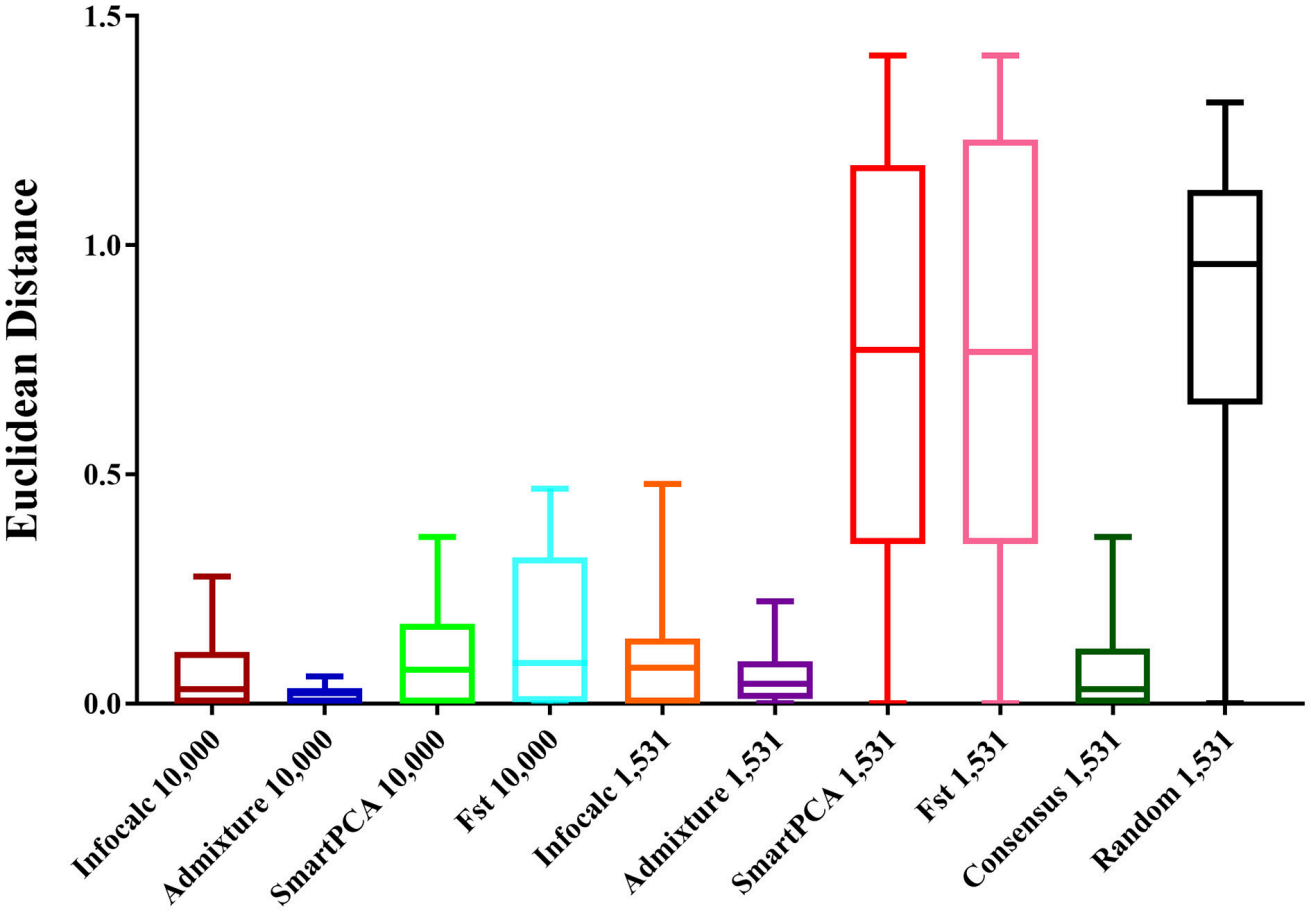
Box and whisker plots comparing the Euclidean distances between the admixture proportions of the gorilla genomes obtained from the CSS and those obtained from the reduced datasets. The number of SNPs the datasets are comprised of is mentioned in their nomenclature. The random dataset was comprised of 1,531 randomly generated SNPs from the CSS and the Consensus-1,531 dataset comprised of 1,531 SNPs that were detected by at least three of the four AIMs-determining approaches.

For comparing the test datasets quantitatively, we computed Euclidean distances between the three admixture components (eastern lowland, Cameroonian and Congolese) of all datasets and the CSS. The shortest mean Euclidean distance (μ = 0.022) was found between Admixture-10,000 and the CSS, closely followed by Infocalc-10,000 and the CSS (μ = 0.064) (Figure 2). Among other 10,000 SNP panels, the longest Euclidean distance was found between the CSS and *F*_*ST*_-10,000, followed by the CSS and SmartPCA-10,000 (0.154 and 0.108, respectively).

Among the 1,531 panels, the shortest distance was revealed between Admixture-1,531 and the CSS (μ = 0.059). Consensus-1,531 appeared as the second most sensitive approach (μ = 0.087), closely followed by Infocalc-1,531 (μ = 0.095). All three aforesaid 1,531 panels highly significantly outperformed all the remaining datasets including the random dataset (Tukey's *post hoc* test; *p*-value < 0.0001). Congruent with our results from qualitative analyses in their inability to capture the accurate population structure for query gorilla genomes, the SmartPCA and *F*_*ST*_-based datasets appeared to be the farthest from the CSS (μ = 0.75 in both cases) and performed similar to the Random-1,531 dataset (Tukey's *post hoc* test; *p*-value = 0.94 and 0.95, respectively). Here further we note that, although Admixture-1,531 had the shortest mean Euclidean distance from the CSS, its performance was statistically very similar to Consensus-1,531 and Infocalc-1,531 (Tukey's *post hoc* test; *p*-value = 0.99).

Overall, our result indicates that while Infocalc-1,531 turned out to be the best method in qualitative ADMIXTURE analysis, Admixture-1,531 was superior to all other approaches in the quantitative analysis. However, in both cases, Consensus-1,531 was a close second and its performance was statistically similar to the other two. Additionally, Consensus-1,531 had discernibly smaller median Euclidean distance from the CSS (0.032) compared to both Infocalc-1,531 (0.078) and Admixture-1,531 (0.043) which further advocates for its candidacy to be considered as the AIMs panel for the gorillas.

### Principal Component Analysis (PCA)

**Figure 3:**
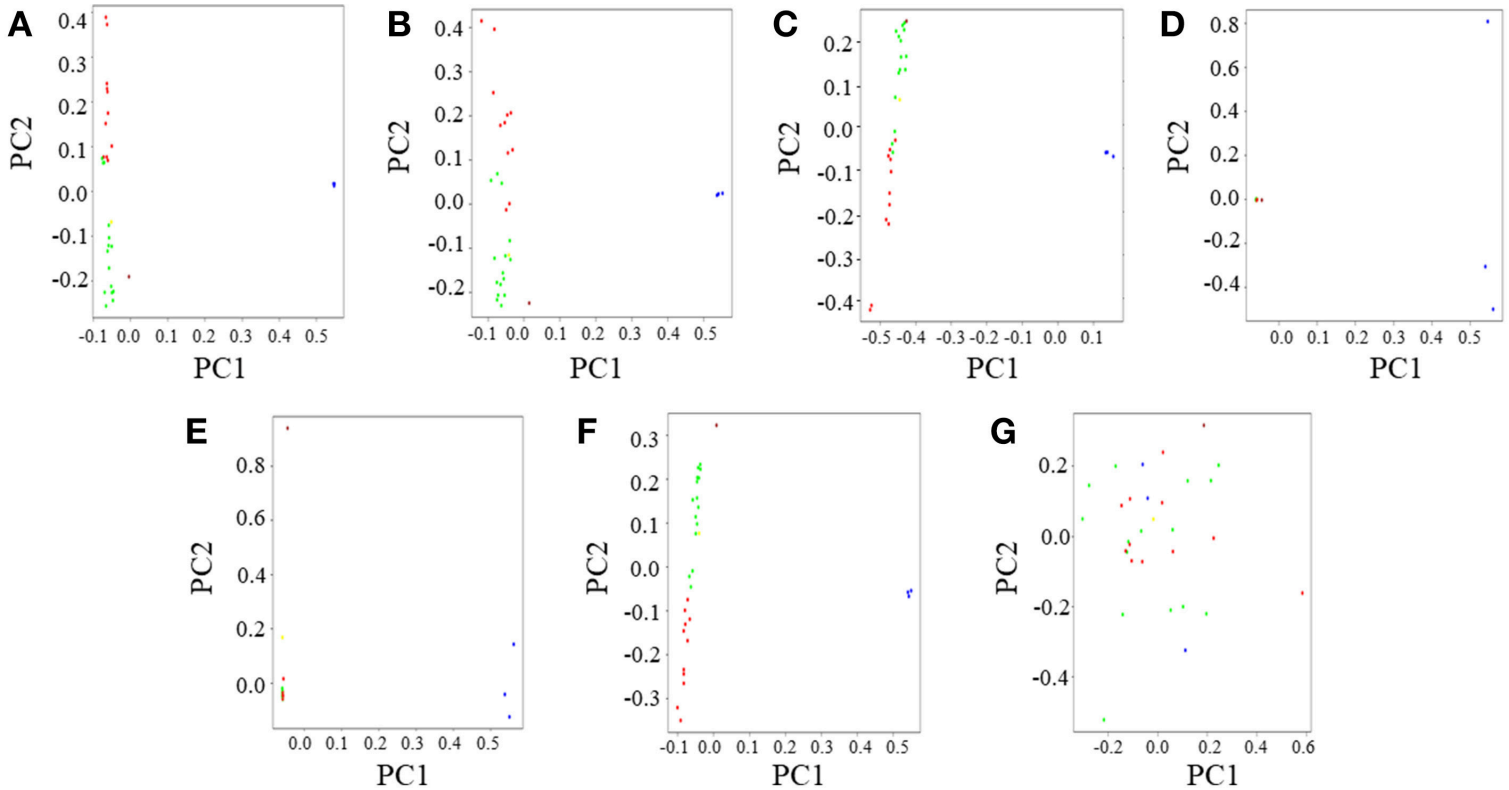
Principal Component Analysis (PCA) of gorilla genomes. PCA plots showing genetic differentiation among query gorilla genomes. The data subsets were generated using top 1,531 most informative SNPs detected through various AIMs determining approaches. **(A)** PCA of the CSS (354,080 SNPs); Here, the X-axis (PC1) explained 45% variance while the Y-axis (PC2) explained 23% variance of the data. **(B)** PCA of Infocalc-1,531; In this case, the X-axis (PC1) explained 45% variance while the Y-axis (PC2) explained only 21% variance of the data. **(C)** PCA of Admixture-1,531; In this case, the X-axis (PC1) explained 68% variance while the Y-axis (PC2) explained 22% variance of the data. **(D)** PCA of SmartPCA-1,531; Here, the X-axis (PC1) explained 88% variance while the Y-axis (PC2) explained only 6% variance of the data. **(E)** PCA of *F*_*ST*_-1,531; In this case, the X-axis (PC1) explained 85% variance while the Y-axis (PC2) explained only 5% variance of the data. **(F)** PCA of Consensus-1,531; In this case, the X-axis (PC1) explained 82% variance while the Y-axis (PC2) explained 10% variance of the data. **(G)** PCA of Random-1,531; Here, the X-axis (PC1) explained 28% variance while the Y-axis (PC2) explained 24% variance of the data. Notable populations are marked with circles such that the blue circles represent eastern lowland gorillas; brown represents the cross river gorilla; and green, red and yellow represents western lowland gorillas of Cameroonian, Congolese and Equatorial Guinean ancestry, respectively. In all cases, PCA was performed in PLINK v1.9 and the top four principal components (PCs) were extracted. Top two PCs (PC1 and PC2), explaining the highest variance of the data were plotted in R v3.2.3.

Principal Component Analysis (PCA) was performed in PLINK v1.9 and the top two PCs were plotted in R v3.2.3. The PCA results for the CSS was in coherence with previous observations of an eastern gorilla-western gorilla contrast along the horizontal principal component (PC1) and vertical delineation (PC2) among western gorilla genomes (Prado-Martinez et al., 2013; Das and Upadhayai, 2018)(Figure 3A, Supplemental Figure S6A). Further, as observed previously, two distinct clusters were found among western gorillas along PC1: one predominantly composed of Cameroonian gorillas and the other predominantly of Congolese gorillas. Also, as found previously, Coco, the only Equatorial Guinea gorilla employed in our study clustered with the Cameroonian gorillas owing to its genomic proximity to the latter (Das and Upadhayai, 2018).

Similar to ADMIXTURE analysis, Infocalc-10,000 (Supplemental Figure S6B) and Admixture-10,000 (Supplemental Figure S6C) best replicated the population clusters depicted by CSS-based dataset (Supplemental Figure S6A) with high precision. Both datasets successfully recapitulated the overlap of some of the Cameroonian and Congolese gorillas at the center of PC2 and the genomic proximity of the cross river gorilla Nyango to Cameroonian gorillas. Among the remaining datasets, SmartPCA-10,000 could recapitulate the overlap of Cameroonian and Congolese gorillas along PC2, but it failed to recapture the high genomic proximity of Nyango with Cameroonian gorillas as depicted by the CSS (Supplemental Figure S6D). Finally, *F*_*ST*_-10,000 portrayed two distinct clusters of Cameroonian and Congolese gorillas and failed to replicate the overlap of some of the Cameroonian and Congolese gorillas at the center of the vertical principal component (PC2) (Supplemental Figure S6E).

Among the 1,531 SNP panels, Infocalc-1,531 was superior to all other AIMs-determining strategies in replicating the population structure of query gorillas depicted by the CSS (Figure 3B). Coherent with the ADMIXTURE analysis, Consensus-1,531 turned out to be the second best (Figure 3F), followed by Admixture-1,531 (Figure 3C). Among the remaining datasets, SmartPCA-1,531 and *F*_*ST*_-1,531 performed discernibly worse and completely failed to depict any contrast among the western gorilla genomes along PC2 (Figures 3D,E). Finally, in concordance with the ADMIXTURE analysis, Random-1,531 was completely unsuccessful in capturing population structure of all query gorillas, such that it even failed to depict the eastern gorilla-western gorilla contrast along the horizontal principal component (PC1) (Figure 3G). The failure of the random dataset once again underscored the superiority of the AIMs over randomly selected markers in portraying population structure of query genomes.

Taking together all analyses, our study revealed that while Infocalc performed better than other approaches in qualitative analysis, the Admixture-based approach turned out to be the best in the quantitative analysis. This indicates that no single AIMs-determining strategy may be sufficient to recapitulate the ancestry information of gorillas. So, we propose that Consensus-1,531 which performed consistently well in both qualitative and quantitative analysis (ranked 2nd in both) should be elucidated as the AIMs panel for the gorillas as it emerged as the smallest set of SNPs that delineates the ancestry information and population structure of gorillas with optimum precision. Further, we have generated a set of 262 most informative SNPs from the 1,531 AIMs panel, which can be detected through common genotyping techniques and are powerful enough to detect fine structure within gorilla populations (Supplemental Table 2).

## Discussion

Over the years, Gorillas, with dwindling population size and increasingly reduced and restricted distribution in the wild, are faced with serious threats for their survival. As a consequence, conservation of wild as well as captive gorillas and preservation of unique gorilla gene pools has garnered a lot of attention in recent years. The gorilla breeding programs that affords to increase genetic diversity in order to avoid inbreeding depression, have been restricted by insufficient information about the ancestry of the gorillas (Wharton, 2009; Nsubuga et al., 2010; Simons et al., 2012; Prado-Martinez et al., 2013). Hence, the determination of the biogeographical affiliation of gorillas can be invaluable to foster their population level (intra-specific) management and preservation of unique gorilla gene pools.

In this study we sought to compare three strategies previously used for AIMs determination, namely Infocalc algorithm (Paschou et al., 2007; Kosoy et al., 2009), Wright's *F*_ST_ (Tian et al., 2007; Kidd et al., 2011; Nievergelt et al., 2013), and Smart Principal Component Analysis (SmartPCA) (Patterson et al., 2006) with a novel ADMIXTURE based approach (Alexander et al., 2009) to delineate an AIMs panel that can reproducibly capture the genomic ancestry of gorillas at the population level and aid in identification of gorillas at the individual level. To this end, we developed the first AIMs panel for gorillas containing 1,531 SNPs that were common to at least three out of four AIMs-determining approaches. Our results indicate that this AIMs panel can recapitulate the ancestry information of query gorillas with high precision and can help in population level identification of gorillas, which can be monumental in the preservation of unique gorilla gene pools and selection of individuals for captive breeding program.

Our AIMs panel (Consensus-1,531) consisted of 1,531 SNPs, generated as a consensus of at least three of the four aforesaid AIMs-determining strategies and thus likely balanced out the limitations of each individual approach (Wilkinson et al., 2011). Here we note that out of 1,531 SNPs, 1,359 SNPs were common among *F*_ST_, ADMIXTURE and SmartPCA and were not detected by the Infocalc based method (Figure 2). The great extent of overlap of top-ranked AIMs of the aforementioned strategies indicates that these three strategies essentially captured the same information regarding the ancestry of query gorillas. Further, while the two worst performing approaches-SmartPCA and *F*_ST_ revealed the highest number of overlapping SNPs (>26%), Infocalc generated the highest number of exclusive SNPs (94%), followed by ADMIXTURE (66%). These results indicates a likely relationship between the exclusiveness of a SNP and its ability to recapture the ancestry information.

Overall, our qualitative and quantitative analyses concur that Consensus-1,531 could recapitulate the ancestry information of query gorillas with high precision. While Consensus-1,531 had the shortest median Euclidean distance from the CSS (0.032), it appeared as the second most sensitive approach in terms of the mean Euclidean distance from the same (μ = 0.087) indicating its high precision of recapitulating the ancestral information depicted by the whole dataset. Further, quantitative assessment reflected that the performance of Consensus-1,531 was indistinct from the larger 10,000 SNP based datasets (*p*-value > 0.99) and had the highest number of individuals (*N* = 9) with zero Euclidean distances from the CSS. However, we note that while Consensus-1,531 successfully replicated the ancestry information of most query gorillas employed in this study, it failed to capture the Cameroonian ancestry component for Carolyn, Delphi and Porta and homogenously assigned them to Congo (Figure 1) and thus appeared to be the second-most sensitive in the qualitative assessment, falling short of the number matched Infocalc derived panel.

Amidst the remaining approaches, we note that *F*_*ST*_ was the poorest in capturing fine-scale population structure of query gorillas, closely followed by the SmartPCA based approach (Figures 1–3), suggesting the ineffectiveness of these two strategies in recapitulating the ancestral history of gorillas. We further note that most AIMs determining approaches employed in this study (except *F*_*ST*_, and SmartPCA) and their consensus appeared to be superior to the randomly selected markers in capturing the population structure delineated by the CSS (Figures 1–3), advocating the usefulness of AIMs in tracing biogeographical origin of organisms over randomized SNPs.

Here we note that the goal of this study was to develop AIMs that can be used to tell apart various populations within western lowland gorilla (below subspecies level). Eastern and western lowland gorillas are considered to be different species and are genetically so distinct from each other that they can be differentiated through most markers present in the complete SNP set (CSS). Despite our restriction in terms of sample size and data availability, since most gorilla genomes used in this study belong to various western gorilla populations (27 out of 31), our results should reflect our intended outcome of deducing AIMs that can differentiate western gorillas below subspecies level.

The quest of developing an AIMs panel for gorillas is not new. A previous study has developed polymorphic MEIs, including those that can be considered ancestry-informative markers and MEIs corresponding to regions of incomplete lineage sorting (ILS) (Hormozdiari et al., 2013). However, to the best of our knowledge, this is the first study to have developed an AIMs panel for gorillas, which can recapitulate their ancestry information with high precision. With limited availability of funding, the conservation geneticists need to draw a balance between the costs of genotyping multiple loci and the inadequacy of information when limited number of loci are genotyped. Comprised of only 1,531 SNPs, the gorilla AIMs panel described here, can become a likely cost-effective solution to this problem. Our AIMs panel can resolve the ancestry information of gorillas with highest resolution power and can detect fine structures within gorilla populations below subspecies level at a highly affordable cost.

## Conclusions

Effective conservation of gorilla populations requires the delineation of their ancestry information to facilitate preservation of the population level integrity of genomic signal and avoidance of inbreeding depression. To this end, we have developed an AIMs panel comprising of 1,531 SNPs that can recapitulate the ancestry information of gorillas with high precision. Our AIMs panel can afford a cost-effective solution to whole genome sequencing and/or large-scale genotyping of gorillas for large-scale biogeographic analysis and conservation genetics studies.

To the best of our knowledge this is the first AIMs panel developed for gorillas that can bolster their efficient management and aid in the conservation of their genetic integrity.

## Author Contributions

RD has conceived the idea of the project, written the manuscript and helped in the analysis. RR and NV performed all the analysis.

### Conflict of Interest Statement

The authors declare that the research was conducted in the absence of any commercial or financial relationships that could be construed as a potential conflict of interest.
