# Supplementary material for: Using Ancestry Informative Markers (AIMs) to Detect Fine Structures Within Gorilla Populations
Source: Front Genet. 2019 Feb 8;10:43. doi: 10.3389/fgene.2019.00043 (PMC6375890; doi:10.3389/fgene.2019.00043)

**Supplemental Table 1**: Biogeographic information about the gorilla individuals employed in current study

| **Species** | **Common name** | **Name** | **Studbook ID** | **Sex** | **Geographic origin according to Studbook[1]** | **Geographic origin according to GPS analysis[2]** |
| --- | --- | --- | --- | --- | --- | --- |
| Western lowland gorilla (*Gorilla gorilla gorilla*) | | Carolyn | 3 | F | Congo | Congo |
|  |  | Abe | 52 | M | Unknown | Cameroon |
|  |  | Porta | 64 | F | Unknown | Congo |
|  |  | Vila | 80 | F | Congo | Cameroon |
|  |  | Helen | 96 | F | Cameroon | Cameroon |
|  |  | Choomba | 180 | F | West Africa | Cameroon |
|  |  | Paki | 191 | F | West Africa | Cameroon |
|  |  | Oko | 192 | F | Unknown | Cameroon |
|  |  | Dolly | 195 | F | Congo | Congo |
|  |  | Delphi | 230 | F | Congo | Congo |
|  |  | Mimi | 241 | F | Cameroon | Cameroon |
|  |  | Banjo | 255 | M | Cameroon | Cameroon |
|  |  | Tzambo | 440 | M | Unknown | Congo |
|  |  | Katie (KB4986) | 498 | F | Unknown | Congo |
|  |  | Katie (B650) | 498 | F | West Africa | Congo |
|  |  | Suzie | 636 | F | Unknown | Cameroon |
|  |  | Kowali | 663 | F | Unknown | Congo |
|  |  | Amani | 899 | F | Unknown | Cameroon |
|  |  | Kolo | 936 | F | Cameroon | Cameroon |
|  |  | Sandra | 969 | F | Cameroon | Cameroon |
|  |  | Kokamo | 1049 | F | Unknown | Congo |
|  |  | Dian | 1091 | F | Cameroon | Cameroon |
|  |  | Bulera | 1120 | F | Cameroon | Congo |
|  |  | Coco | 1351 | F | Equatorial Guinea | Equatorial Guinea |
|  |  | Azizi | 1459 | M | Cameroon | Cameroon |
|  |  | Akiba Beri | 1926 | F | Cameroon | Cameroon |
|  |  | Anthal | 1930 | F | Cameroon | Cameroon |
| Eastern lowland gorilla (*Gorilla beringei graueri*) | | M'kubwa | 9907 | M | DRC - Tulakwa, Northwest of Bukavu | DRC |
|  |  | Kaisi | 9909 | M | DRC - Walikale region, Nord-Kivu | DRC |
|  |  | Victoria | 9919 | F | DRC | DRC |
| Gorilla gorilla diehli | Cross River gorilla | Nyango | 9941 | F | Cameroon | - |

1. Prado-Martinez J, Sudmant PH, Kidd JM, Li H, Kelley JL, Lorente-Galdos B, Veeramah KR, Woerner AE, O'Connor TD, Santpere G *et al*: **Great ape genetic diversity and population history**. *Nature* 2013, **499**(7459):471-475.

2. Das R, Upadhayai P: **Application of the Geographic Population Structure (GPS) algorithm for biogeographical analyses of non-human individuals: a case study of wild and captive gorillas**. *BMC Bioinformatics* 2018, **In Press**.

**Supplemental Table 2:** Minor Allele Frequencies (MAF) of the top 264 most informative AIMs

| **CHR** | **SNP** | **A1** | **A2** | **MAF** | **NCHROBS** |
| --- | --- | --- | --- | --- | --- |
| 1 | chr1:10825999 | C | T | 0.08065 | 62 |
| 1 | chr1:18865422 | T | C | 0.08065 | 62 |
| 1 | chr1:70664857 | G | T | 0.08065 | 62 |
| 1 | chr1:70865657 | G | A | 0.08065 | 62 |
| 1 | chr1:72853600 | A | G | 0.08065 | 62 |
| 1 | chr1:76975666 | C | T | 0.08065 | 62 |
| 1 | chr1:83833062 | A | T | 0.08065 | 62 |
| 1 | chr1:90376974 | C | A | 0.08065 | 62 |
| 1 | chr1:94368990 | A | G | 0.08065 | 62 |
| 1 | chr1:100033015 | C | T | 0.08065 | 62 |
| 1 | chr1:104190707 | G | A | 0.08065 | 62 |
| 1 | chr1:104203772 | C | T | 0.08065 | 62 |
| 1 | chr1:106035157 | A | G | 0.08065 | 62 |
| 1 | chr1:159086407 | G | C | 0.08065 | 62 |
| 1 | chr1:169548629 | T | C | 0.08065 | 62 |
| 1 | chr1:180558202 | C | T | 0.08065 | 62 |
| 1 | chr1:185332668 | A | G | 0.08065 | 62 |
| 1 | chr1:186362233 | G | A | 0.08065 | 62 |
| 1 | chr1:189374641 | A | T | 0.06667 | 60 |
| 1 | chr1:200908252 | C | T | 0.08065 | 62 |
| 1 | chr1:219562569 | A | G | 0.08065 | 62 |
| 1 | chr1:223766984 | A | C | 0.08065 | 62 |
| 1 | chr1:223897405 | T | C | 0.08065 | 62 |
| 1 | chr1:223926680 | T | C | 0.08065 | 62 |
| 1 | chr1:224395504 | A | G | 0.08065 | 62 |
| 1 | chr1:224409489 | T | C | 0.08065 | 62 |
| 1 | chr1:224565390 | A | G | 0.08065 | 62 |
| 1 | chr1:231784452 | G | A | 0.08065 | 62 |
| 1 | chr1:239919773 | C | T | 0.08333 | 60 |
| 1 | chr1:244130607 | G | A | 0.08065 | 62 |
| 2 | chr2:2353148 | T | A | 0.08065 | 62 |
| 2 | chr2:9445320 | C | T | 0.08065 | 62 |
| 2 | chr2:27632203 | T | G | 0.08065 | 62 |
| 2 | chr2:69431411 | T | C | 0.08065 | 62 |
| 2 | chr2:69445355 | A | G | 0.08065 | 62 |
| 2 | chr2:69503022 | A | G | 0.08065 | 62 |
| 2 | chr2:126665658 | C | T | 0.08065 | 62 |
| 2 | chr2:133656590 | T | A | 0.08065 | 62 |
| 2 | chr2:137450705 | A | G | 0.08065 | 62 |
| 2 | chr2:137464578 | T | C | 0.08065 | 62 |
| 2 | chr2:139982481 | A | T | 0.08333 | 60 |
| 2 | chr2:151058467 | G | T | 0.09677 | 62 |
| 2 | chr2:171902573 | A | G | 0.08065 | 62 |
| 2 | chr2:176487102 | C | A | 0.08065 | 62 |
| 2 | chr2:176597159 | C | A | 0.08065 | 62 |
| 2 | chr2:178448749 | C | T | 0.08065 | 62 |
| 2 | chr2:178481619 | A | G | 0.08065 | 62 |
| 2 | chr2:195660442 | T | C | 0.08065 | 62 |
| 2 | chr2:204696154 | A | G | 0.08065 | 62 |
| 3 | chr3:4222148 | T | C | 0.08065 | 62 |
| 3 | chr3:8044463 | T | C | 0.08065 | 62 |
| 3 | chr3:13849118 | C | T | 0.08065 | 62 |
| 3 | chr3:20360621 | A | T | 0.08333 | 60 |
| 3 | chr3:21017432 | G | C | 0.08065 | 62 |
| 3 | chr3:24069203 | T | C | 0.08065 | 62 |
| 3 | chr3:62747279 | A | G | 0.08065 | 62 |
| 3 | chr3:72771216 | A | T | 0.08065 | 62 |
| 3 | chr3:75130241 | G | T | 0.08065 | 62 |
| 3 | chr3:75263080 | G | A | 0.08065 | 62 |
| 3 | chr3:107117400 | G | A | 0.08065 | 62 |
| 3 | chr3:110789120 | A | G | 0.08065 | 62 |
| 3 | chr3:156319184 | G | A | 0.08065 | 62 |
| 3 | chr3:167140870 | G | A | 0.08065 | 62 |
| 3 | chr3:177086808 | T | C | 0.08065 | 62 |
| 3 | chr3:181767154 | G | A | 0.08065 | 62 |
| 4 | chr4:12495420 | A | G | 0.08065 | 62 |
| 4 | chr4:38322243 | T | C | 0.08065 | 62 |
| 4 | chr4:54207240 | A | G | 0.08065 | 62 |
| 4 | chr4:63414904 | T | C | 0.08065 | 62 |
| 4 | chr4:63828881 | C | T | 0.08065 | 62 |
| 4 | chr4:64777821 | C | T | 0.08065 | 62 |
| 4 | chr4:64784635 | C | T | 0.08065 | 62 |
| 4 | chr4:71779826 | T | C | 0.08065 | 62 |
| 4 | chr4:93910506 | G | A | 0.08065 | 62 |
| 4 | chr4:96331766 | T | C | 0.08065 | 62 |
| 4 | chr4:105288306 | G | A | 0.08065 | 62 |
| 4 | chr4:116731686 | G | A | 0.08065 | 62 |
| 4 | chr4:130484483 | G | A | 0.08065 | 62 |
| 4 | chr4:130618818 | C | T | 0.08065 | 62 |
| 4 | chr4:135108477 | A | G | 0.08065 | 62 |
| 4 | chr4:135124952 | C | G | 0.08065 | 62 |
| 4 | chr4:135425608 | C | G | 0.08333 | 60 |
| 4 | chr4:170142836 | T | C | 0.08065 | 62 |
| 4 | chr4:170154585 | T | C | 0.08065 | 62 |
| 4 | chr4:179745040 | A | G | 0.08065 | 62 |
| 4 | chr4:186069938 | G | A | 0.08065 | 62 |
| 4 | chr4:186597044 | T | C | 0.08065 | 62 |
| 5 | chr5:4907970 | C | T | 0.08065 | 62 |
| 5 | chr5:7740494 | C | T | 0.08065 | 62 |
| 5 | chr5:19679329 | A | G | 0.08065 | 62 |
| 5 | chr5:20098447 | G | A | 0.08065 | 62 |
| 5 | chr5:27566772 | C | T | 0.08065 | 62 |
| 5 | chr5:27577717 | T | C | 0.08065 | 62 |
| 5 | chr5:30324805 | A | G | 0.08065 | 62 |
| 5 | chr5:43180240 | A | G | 0.08065 | 62 |
| 5 | chr5:43551678 | T | C | 0.08065 | 62 |
| 5 | chr5:43578802 | A | G | 0.08065 | 62 |
| 5 | chr5:61955344 | T | C | 0.08065 | 62 |
| 5 | chr5:62176487 | A | G | 0.08065 | 62 |
| 5 | chr5:70838486 | A | G | 0.08065 | 62 |
| 5 | chr5:71563204 | C | T | 0.08065 | 62 |
| 5 | chr5:75726858 | A | G | 0.08065 | 62 |
| 5 | chr5:83029467 | G | T | 0.08065 | 62 |
| 5 | chr5:85806881 | G | A | 0.08065 | 62 |
| 5 | chr5:85819681 | A | G | 0.08065 | 62 |
| 5 | chr5:100885489 | C | T | 0.08065 | 62 |
| 5 | chr5:115778178 | C | A | 0.08065 | 62 |
| 5 | chr5:127146762 | A | T | 0.06667 | 60 |
| 5 | chr5:132113042 | T | C | 0.08065 | 62 |
| 5 | chr5:132198447 | T | G | 0.08065 | 62 |
| 5 | chr5:132250392 | A | G | 0.08065 | 62 |
| 5 | chr5:162490460 | C | A | 0.08065 | 62 |
| 5 | chr5:166632845 | G | A | 0.08065 | 62 |
| 5 | chr5:175813932 | A | G | 0.08065 | 62 |
| 6 | chr6:17161213 | T | C | 0.08065 | 62 |
| 6 | chr6:17178128 | C | G | 0.08065 | 62 |
| 6 | chr6:22881649 | A | G | 0.08065 | 62 |
| 6 | chr6:29756532 | T | C | 0.08333 | 60 |
| 6 | chr6:29767753 | G | A | 0.08065 | 62 |
| 6 | chr6:31119821 | A | G | 0.08065 | 62 |
| 6 | chr6:31127822 | A | G | 0.08065 | 62 |
| 6 | chr6:39323587 | T | C | 0.08065 | 62 |
| 6 | chr6:54990378 | C | T | 0.08065 | 62 |
| 6 | chr6:77414605 | C | T | 0.08065 | 62 |
| 6 | chr6:80899751 | C | T | 0.08065 | 62 |
| 6 | chr6:84537398 | T | C | 0.08333 | 60 |
| 6 | chr6:85549759 | C | T | 0.08065 | 62 |
| 6 | chr6:94226198 | G | T | 0.08065 | 62 |
| 6 | chr6:99513624 | C | T | 0.1129 | 62 |
| 6 | chr6:111643697 | G | T | 0.08065 | 62 |
| 6 | chr6:120187864 | T | C | 0.08065 | 62 |
| 6 | chr6:121995561 | A | G | 0.08065 | 62 |
| 6 | chr6:146003471 | T | C | 0.08065 | 62 |
| 6 | chr6:147723584 | C | A | 0.08065 | 62 |
| 6 | chr6:159212583 | A | T | 0.08065 | 62 |
| 6 | chr6:163999892 | G | A | 0.08065 | 62 |
| 7 | chr7:3945675 | G | A | 0.08065 | 62 |
| 7 | chr7:67509210 | A | G | 0.08065 | 62 |
| 7 | chr7:90440612 | C | T | 0.08065 | 62 |
| 7 | chr7:90456975 | A | G | 0.08065 | 62 |
| 7 | chr7:91553585 | G | A | 0.08065 | 62 |
| 7 | chr7:97959029 | G | A | 0.08065 | 62 |
| 7 | chr7:113977696 | C | A | 0.08065 | 62 |
| 7 | chr7:122821043 | T | C | 0.08065 | 62 |
| 7 | chr7:134640768 | A | C | 0.08065 | 62 |
| 7 | chr7:138312656 | A | G | 0.08065 | 62 |
| 7 | chr7:142694311 | C | A | 0.08065 | 62 |
| 8 | chr8:2923572 | A | G | 0.08333 | 60 |
| 8 | chr8:14857108 | G | T | 0.08065 | 62 |
| 8 | chr8:37335036 | G | A | 0.08065 | 62 |
| 8 | chr8:37341056 | G | A | 0.08065 | 62 |
| 8 | chr8:40923566 | G | A | 0.08065 | 62 |
| 8 | chr8:51540869 | C | T | 0.08065 | 62 |
| 8 | chr8:52584215 | A | G | 0.08333 | 60 |
| 8 | chr8:52729218 | T | C | 0.08065 | 62 |
| 8 | chr8:56066671 | A | T | 0.08065 | 62 |
| 8 | chr8:64088399 | A | G | 0.08065 | 62 |
| 8 | chr8:69154106 | G | A | 0.08065 | 62 |
| 8 | chr8:92130161 | C | T | 0.08065 | 62 |
| 8 | chr8:92144492 | C | T | 0.08065 | 62 |
| 8 | chr8:92161430 | C | A | 0.08065 | 62 |
| 8 | chr8:92199207 | T | C | 0.08065 | 62 |
| 8 | chr8:92209053 | T | C | 0.08065 | 62 |
| 8 | chr8:92221969 | A | G | 0.08065 | 62 |
| 8 | chr8:92247458 | G | A | 0.08065 | 62 |
| 8 | chr8:92258773 | T | C | 0.08065 | 62 |
| 8 | chr8:96835279 | T | C | 0.08065 | 62 |
| 8 | chr8:115352528 | C | T | 0.08065 | 62 |
| 8 | chr8:115370391 | T | C | 0.08621 | 58 |
| 8 | chr8:129676721 | A | G | 0.08065 | 62 |
| 8 | chr8:143488888 | T | G | 0.08333 | 60 |
| 9 | chr9:8673692 | C | G | 0.08065 | 62 |
| 9 | chr9:16276303 | A | G | 0.08065 | 62 |
| 9 | chr9:98294226 | G | C | 0.08333 | 60 |
| 9 | chr9:98446357 | G | A | 0.08065 | 62 |
| 9 | chr9:100948052 | A | G | 0.08065 | 62 |
| 9 | chr9:100966420 | C | A | 0.08065 | 62 |
| 9 | chr9:101042757 | A | G | 0.08065 | 62 |
| 9 | chr9:111856539 | A | G | 0.08065 | 62 |
| 9 | chr9:112560548 | A | G | 0.08065 | 62 |
| 10 | chr10:6254949 | T | C | 0.08065 | 62 |
| 10 | chr10:22740601 | G | A | 0.08065 | 62 |
| 10 | chr10:34547121 | T | C | 0.08065 | 62 |
| 10 | chr10:68094874 | A | C | 0.08333 | 60 |
| 10 | chr10:76612667 | T | C | 0.08065 | 62 |
| 10 | chr10:77829603 | C | T | 0.08065 | 62 |
| 10 | chr10:106371977 | C | T | 0.08065 | 62 |
| 11 | chr11:23937824 | A | G | 0.08065 | 62 |
| 11 | chr11:27786783 | C | T | 0.08065 | 62 |
| 11 | chr11:28168221 | G | T | 0.08065 | 62 |
| 11 | chr11:42044402 | T | C | 0.08065 | 62 |
| 11 | chr11:46886537 | T | C | 0.08065 | 62 |
| 11 | chr11:56337455 | T | A | 0.08065 | 62 |
| 11 | chr11:74018785 | G | A | 0.08065 | 62 |
| 11 | chr11:91953139 | G | A | 0.08065 | 62 |
| 11 | chr11:93255488 | A | C | 0.08065 | 62 |
| 11 | chr11:93391269 | A | G | 0.09677 | 62 |
| 11 | chr11:104175751 | T | C | 0.08065 | 62 |
| 11 | chr11:110075998 | C | T | 0.08065 | 62 |
| 11 | chr11:115141355 | A | G | 0.08065 | 62 |
| 11 | chr11:129627760 | G | C | 0.08065 | 62 |
| 11 | chr11:129647874 | C | T | 0.08065 | 62 |
| 11 | chr11:129671834 | T | G | 0.08065 | 62 |
| 11 | chr11:129696525 | A | G | 0.08065 | 62 |
| 11 | chr11:129716586 | T | C | 0.08065 | 62 |
| 11 | chr11:132134497 | T | C | 0.08065 | 62 |
| 11 | chr11:133123860 | A | G | 0.08065 | 62 |
| 12 | chr12:1577445 | G | A | 0.08065 | 62 |
| 12 | chr12:21184795 | G | A | 0.08333 | 60 |
| 12 | chr12:50838263 | C | A | 0.08065 | 62 |
| 12 | chr12:50994609 | C | G | 0.08065 | 62 |
| 12 | chr12:91264264 | G | T | 0.08333 | 60 |
| 12 | chr12:101270186 | A | G | 0.08065 | 62 |
| 13 | chr13:24264660 | G | A | 0.08065 | 62 |
| 13 | chr13:24295268 | G | A | 0.08065 | 62 |
| 13 | chr13:70376115 | G | T | 0.08065 | 62 |
| 13 | chr13:81410952 | A | G | 0.08065 | 62 |
| 13 | chr13:84421082 | C | T | 0.08065 | 62 |
| 14 | chr14:46304453 | C | T | 0.08065 | 62 |
| 14 | chr14:57799812 | T | C | 0.08065 | 62 |
| 14 | chr14:57851328 | G | A | 0.08065 | 62 |
| 14 | chr14:57947114 | C | T | 0.08065 | 62 |
| 14 | chr14:69005540 | A | G | 0.08065 | 62 |
| 14 | chr14:91262805 | G | T | 0.08065 | 62 |
| 14 | chr14:102574137 | A | T | 0.08333 | 60 |
| 15 | chr15:38595385 | T | C | 0.09677 | 62 |
| 15 | chr15:38713920 | C | A | 0.08621 | 58 |
| 15 | chr15:43966455 | T | C | 0.08065 | 62 |
| 15 | chr15:52748809 | T | A | 0.08065 | 62 |
| 15 | chr15:53428336 | A | T | 0.08065 | 62 |
| 15 | chr15:76197341 | G | A | 0.08065 | 62 |
| 16 | chr16:24836059 | C | T | 0.08065 | 62 |
| 16 | chr16:50823626 | C | A | 0.08065 | 62 |
| 16 | chr16:67790699 | T | C | 0.08065 | 62 |
| 16 | chr16:67821105 | A | G | 0.08065 | 62 |
| 16 | chr16:79655208 | A | G | 0.08065 | 62 |
| 16 | chr16:79983598 | G | A | 0.08065 | 62 |
| 16 | chr16:86148627 | A | G | 0.08065 | 62 |
| 17 | chr17:17855366 | T | C | 0.08333 | 60 |
| 17 | chr17:18035716 | T | C | 0.08065 | 62 |
| 17 | chr17:28621901 | T | G | 0.08065 | 62 |
| 17 | chr17:45543464 | A | G | 0.08065 | 62 |
| 17 | chr17:57399776 | C | A | 0.08065 | 62 |
| 17 | chr17:63126770 | T | C | 0.08065 | 62 |
| 17 | chr17:75584778 | C | T | 0.08065 | 62 |
| 18 | chr18:5415846 | T | G | 0.08065 | 62 |
| 18 | chr18:11131657 | A | G | 0.08065 | 62 |
| 18 | chr18:31967420 | C | T | 0.08333 | 60 |
| 18 | chr18:32001703 | T | C | 0.08065 | 62 |
| 18 | chr18:41087314 | T | C | 0.08065 | 62 |
| 19 | chr19:19053851 | A | G | 0.1167 | 60 |
| 19 | chr19:19083579 | T | C | 0.09677 | 62 |
| 19 | chr19:38725478 | G | A | 0.08065 | 62 |
| 19 | chr19:52782942 | T | C | 0.08065 | 62 |
| 20 | chr20:10796225 | A | T | 0.08065 | 62 |
| 20 | chr20:13444362 | T | A | 0.08065 | 62 |
| 20 | chr20:52100923 | G | C | 0.08065 | 62 |
| 21 | chr21:20113053 | T | C | 0.08065 | 62 |
| 21 | chr21:29456744 | A | G | 0.08065 | 62 |
| 21 | chr21:45631132 | T | C | 0.08065 | 62 |
| 22 | chr22:24427753 | T | C | 0.08065 | 62 |
| 22 | chr22:40710400 | A | G | 0.08333 | 60 |

**Supplemental fig. S1.** Distribution of Infocalc metric scores for all 354,080 SNPs employed in this study. The distribution of infocalc metric scores for top 10,000 most informative markers is shown in the inset.


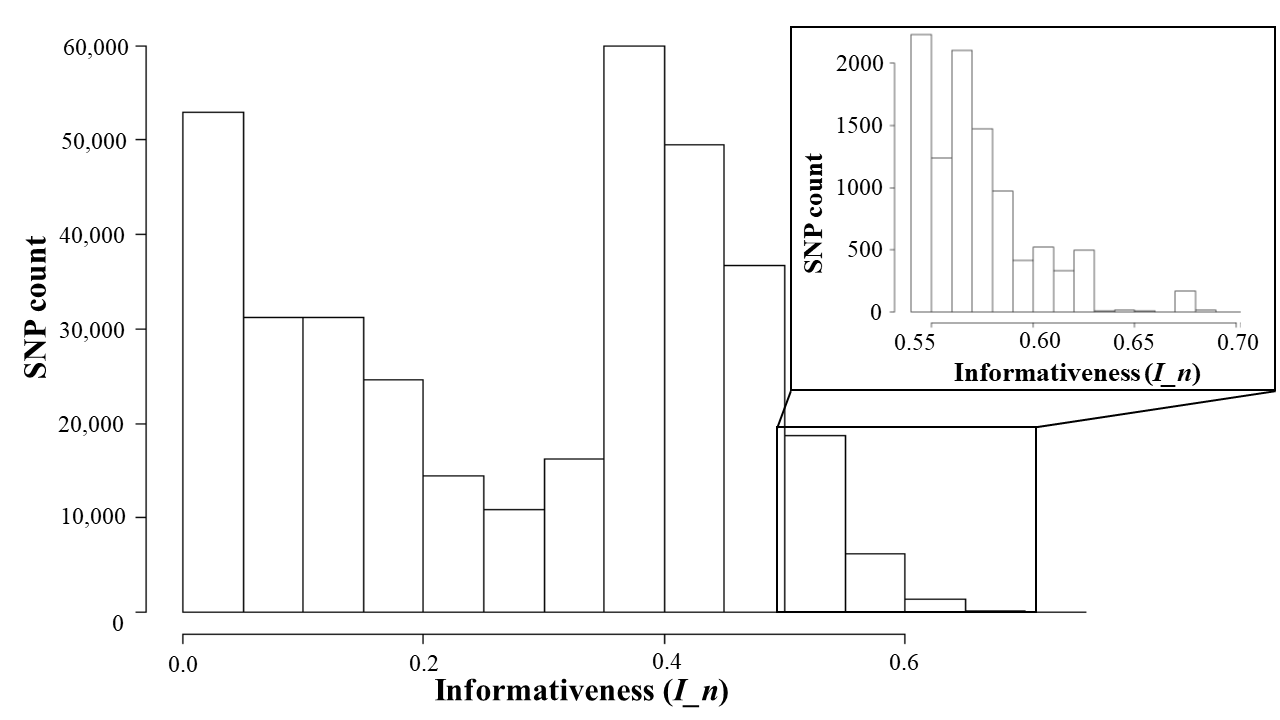


**Supplemental fig. S2.** Distribution of *F_ST_* values for all 354,080 SNPs employed in this study. The distribution of *F_ST_* values for top 10,000 most informative markers is shown in the inset.

**
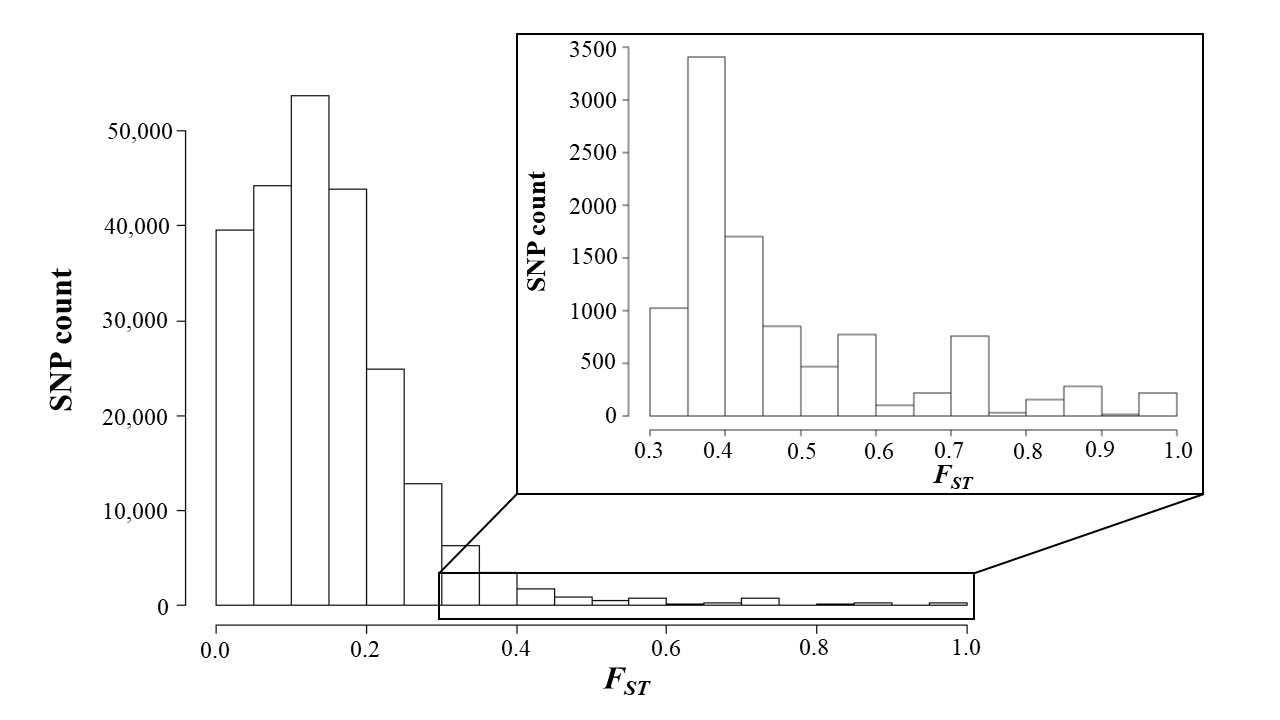
**

**Supplemental fig. S3.** Distribution of weightage scores (for PC1) for all 354,080 SNPs employed in this study. The distribution of weightage scores (for PC1) for top 10,000 most informative markers is shown in the inset.

**
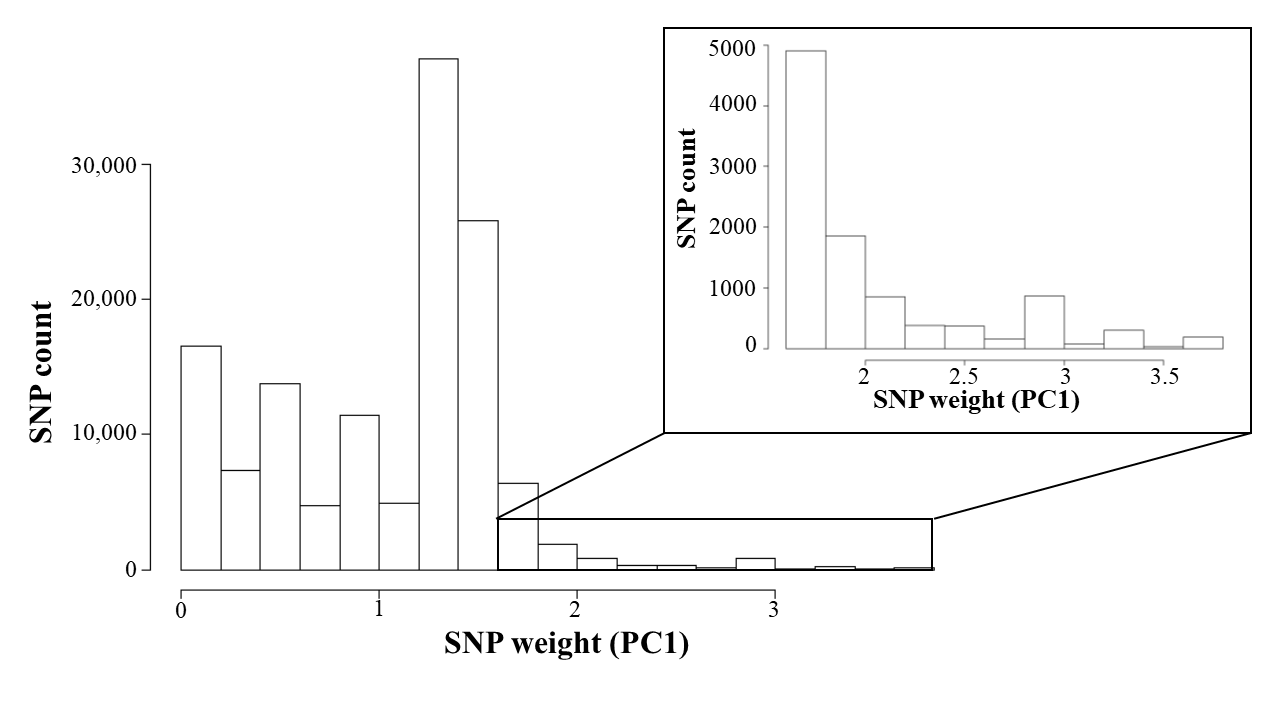
**

**Supplemental fig. S4.** Venn diagram showing the extent of overlap of the top 10,000 AIMs detected by various AIMs-determining approaches.


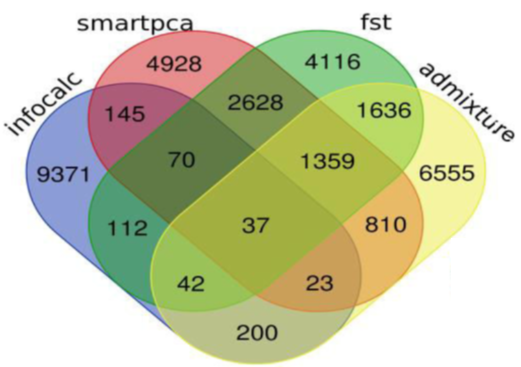


**Supplemental fig. S5. Admixture analysis of data subsets generated through informative SNPs inferred using various AIMs-determining strategies.** Admixture plots showing the ancestry components of South Asian genomes. (A) Admixture analysis of the dataset generated through Complete SNP set (CSS) (354,080 SNPs); (B) Admixture analysis of dataset generated through top 10,000 Infocalc-detected SNPs; (C) Admixture analysis of dataset generated through 10,662 ADMIXTURE-detected SNPs; (D) Admixture analysis of dataset generated through top 10,000 SmartPCA-detected SNPs; and (E) Admixture analysis of dataset generated through top 10,000 *F_ST_-*detected SNPs. Admixture proportions were generated through an unsupervised admixture analysis at *K*=3 using ADMIXTURE v1.3 and plotted in R v3.2.3. Each individual is represented by a vertical line partitioned into colored segments whose lengths are proportional to the contributions of the ancestral components to the genome of the individual. Blue represents eastern lowland ancestry component while green and red represent Cameroonian and Congolese ancestral components respectively.


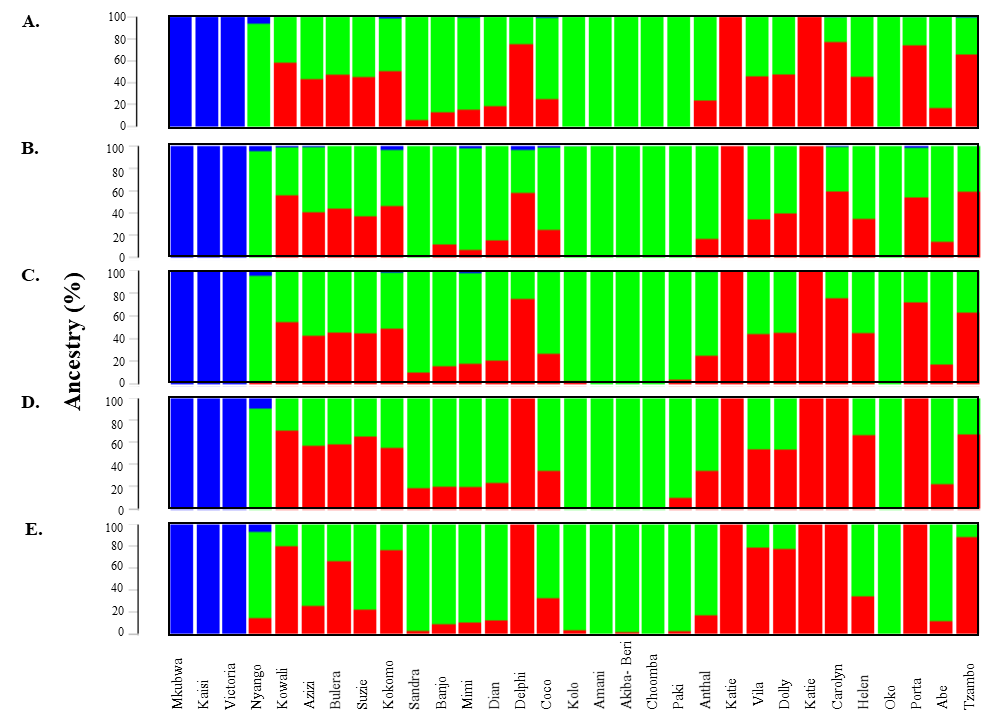


**Supplemental fig. S6. Principal component analysis (PCA) of gorilla genomes** **employing 10,000 SNP based datasets**. PCA plot showing genetic differentiation among South Asian genomes. (A) PCA of the CSS (354,080 SNPs); Here, the X-axis (PC1) explained 45% variance while the Y-axis (PC2) explained 23% variance of the data. (B) PCA of Infocalc-10,000; In this case, the X-axis (PC1) explained 45% variance while the Y-axis (PC2) explained 22% variance of the data. (C) PCA of Admixture-10,000; In this case, the X-axis (PC1) explained 67% variance while the Y-axis (PC2) explained 21% variance of the data. (D) PCA of SmartPCA-10,000; In this case, the X-axis (PC1) explained 83% variance while the Y-axis (PC2) explained only 7% variance of the data. (E) PCA of *F_ST_-*10,000; Here, the X-axis (PC1) explained 54% variance while the Y-axis (PC2) explained 22% variance of the data. Notable populations are marked with circles such that blue circles represent eastern lowland gorillas; brown represents the cross river gorilla; and green, red and yellow represents western lowland gorillas with Cameroonian, Congolese and Equatorial Guinean ancestry respectively. In all cases, PCA was performed in PLINK v1.9 and the top four principal components (PCs) were extracted. Top two PCs (PC1 and PC2), explaining the highest variance of the data were plotted in R v3.2.3.


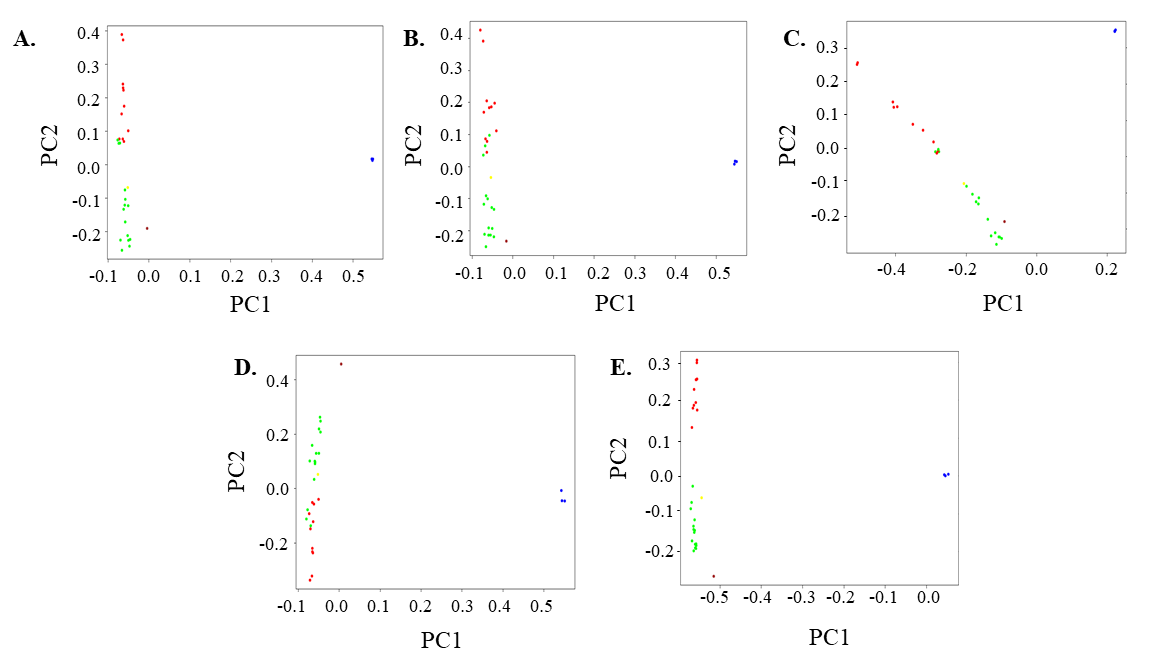

Supplement: Supplementary file 1 [file Data_Sheet_1.docx]
